# Successful management of synchronous prostate adenocarcinoma and lung squamous cell carcinoma with sintilimab and ADT in a frail elderly patient with bronchiectasis: a case report

**DOI:** 10.3389/fimmu.2026.1845049

**Published:** 2026-10-01

**Authors:** Yuerong Huang, Wanlin Zhan, Tingfeng Su, Ying Liu

**Affiliations:** Department of Oncology, People’s Hospital of Huadu District, Guangzhou, Guangdong, China

**Keywords:** androgen deprivation therapy, bronchiectasis, comprehensive geriatric assessment, frailty, multiple primary malignant neoplasms, PD-L1 negative, sintilimab, synchronous dual cancer

## Abstract

**Background:**

The management of elderly patients with multiple primary malignant neoplasms (MPMNs) presents a significant clinical challenge, requiring a balance between oncological efficacy and treatment tolerability. This case report details an elderly, non-verbal male with significant cardiopulmonary comorbidities who presented with synchronous prostate adenocarcinoma and lung squamous cell carcinoma (LUSC). The coexistence of two primary malignancies complicated by recurrent pulmonary infection is exceedingly rare in both clinical guidelines and real-world practice, making the proposed treatment strategy particularly valuable for clinical reference.

**Case presentation:**

A 74-year-old congenitally deaf-mute male with high frailty risk, complicated by hypertension, coronary heart disease and bronchiectasis with chronic Pseudomonas aeruginosa colonization, was diagnosed with synchronous prostate adenocarcinoma and lung squamous cell carcinoma (LUSC). In the available archival pathology report, immunohistochemical testing used a PD-1 mouse monoclonal antibody (clone MX033; Fuzhou Maixin Biotechnology Co., Ltd.) and showed PD-1 positivity in no more than 1% of infiltrating lymphocytes. This result does not measure PD-L1 expression on tumor cells and does not provide a tumor proportion score (TPS); the original 2024 slides were archived and no tumor-cell PD-L1 assay/TPS result was available for re-evaluation. Baseline comprehensive geriatric assessment (CGA) confirmed high chemotherapy toxicity risk, and the patient’s family declined platinum-doublet intensive chemotherapy. Initial combined androgen deprivation therapy (ADT) for prostate cancer plus afatinib first-line targeted therapy for LUSC failed to control the disease, followed by progressive disease after second-line single-agent vinorelbine. Subsequently, standard-dose sintilimab (PD-1 inhibitor) monotherapy was administered as third-line treatment for LUSC with continuous ADT maintained for prostate cancer.

**Results:**

Serial radiology revealed durable marked regression of lung lesions and sustained stable prostate disease, achieving synchronous dual-tumor control. Sintilimab was prescribed on a q21-day schedule but was not delivered uninterruptedly: 12 administrations were given between December 24, 2024 and June 9, 2026, with infection-related interruptions, including a longest interval of more than 3 months. Recurrent bronchiectasis exacerbations with Pseudomonas aeruginosa infection were managed by culture-directed antibiotics and temporary interruption rather than permanent cessation of immunotherapy. No grade 2 or higher immune-related adverse events were documented during the reported follow-up, and the patient’s clinical condition and tumor status were stable at the latest recorded follow-up.

**Conclusion:**

This single case describes the clinical feasibility of standard-dose sintilimab plus ongoing ADT for synchronous prostate adenocarcinoma and LUSC in a frail elderly patient who declined platinum-doublet intensive chemotherapy. The observed response occurred in the context of intermittent, infection-related treatment delays and should not be interpreted as proof of a causal immune-endocrine mechanism. Because tumor-cell PD-L1 TPS was unavailable, biomarker-based conclusions cannot be drawn. This hypothesis-generating observation may inform future studies and individualized decision-making in similarly complex patients.

## 1 Introduction

The occurrence of multiple primary malignant neoplasms (MPMNs) in a single patient, defined as two or more histologically distinct primary cancers, presents a significant clinical challenge, particularly in the elderly population (1). The rising incidence of MPMNs is attributed to factors including genetic predisposition, environmental exposures, and improved diagnostic capabilities leading to longer patient survival (2). Prostate adenocarcinoma and lung squamous cell carcinoma (LUSC) are both prevalent malignancies in older men, yet their co-occurrence as synchronous primaries is relatively uncommon (1). Current guidelines for managing synchronous dual malignancies are largely extrapolated from single-tumor evidence, leaving a substantial clinical gap regarding optimal combination strategies that simultaneously address both primaries without cumulative toxicity. In particular, the integration of immunotherapy with endocrine therapy for synchronous prostate and lung cancers lacks unified guideline recommendations, and existing literature is limited to isolated case reports and small retrospective series (3). LUSC is notoriously difficult to treat in the advanced setting due to a paucity of actionable driver mutations, rendering targeted therapies like EGFR inhibitors largely ineffective, as evidenced by their low objective response rates and short progression-free survival (4). In this archival case, a tumor-cell PD-L1 TPS was not available; therefore, any relationship between PD-L1 status and treatment response cannot be assessed.

This case presents a patient belonging to a particularly vulnerable subgroup rarely captured in existing reports. The patient was an elderly, non-verbal individual with significant cardiopulmonary comorbidities, including hypertension, coronary artery disease, and bronchiectasis with chronic Pseudomonas aeruginosa airway colonization, whose family declined conventional platinum-doublet intensive chemotherapy. The superimposition of three high-risk factors — advanced age with frailty, congenital deaf-mutism precluding autonomous communication, and long-standing structural lung disease — is exceedingly rare in published literature. This compound vulnerability makes the present case uniquely valuable, as it represents a population for which no established treatment paradigm exists. Standardized comprehensive geriatric assessment (CGA) objectively confirmed high frailty and chemotherapy toxicity risk, supporting a shared decision to avoid platinum-doublet intensive chemotherapy (5). Additional published evidence relevant to MPMNs, prostate cancer biology and immunomodulation, bronchiectasis, geriatric treatment tolerability, PD-L1 assessment, synchronous primary cancers, and infection control is cited in references (6–15).

These compounded clinical dilemmas raise a critical unresolved question: For a frail elderly patient with synchronous dual primary malignancies (prostate adenocarcinoma and LUSC), who has a high predicted toxicity risk and whose family declined platinum-doublet intensive chemotherapy, and who suffers from bronchiectasis complicated by recurrent Pseudomonas aeruginosa infection, how can a low-toxicity combined regimen with non-overlapping adverse effects be selected alongside a standardized infection intervention protocol to support ongoing, but potentially interrupted, immunotherapy?

This case report addresses the above question through standard-dose sintilimab monotherapy combined with persistent androgen deprivation strategy, paired with tiered anti-infective supportive care. Serial CGA was performed to stratify treatment tolerance throughout the whole course. The entire case presentation, efficacy evaluation, discussion, and conclusion are organized around this core clinical question. Notably, in clinical practice, geriatric patients frequently receive reduced doses of chemotherapy or immunotherapy in an attempt to lower adverse reactions, while this strategy remains controversial and lacks sufficient clinical evidence. In the present case, sintilimab was administered at the standard dose without dose reduction; however, treatment delivery was intermittent because of recurrent pulmonary infection. Since the patient was congenitally deaf-mute and unable to communicate independently, all medical decisions were made by his family as surrogates based on the patient’s best interests. This case contributes hypothesis-generating insights into individualized management of complex MPMNs.

## 2 Case presentation

### 2.1 Patient information

A 74-year-old congenitally deaf-mute male presented to the emergency department on May 2, 2024, with a 4-day history of chest pain, left shoulder pain, and persistent cough, and was subsequently admitted to the oncology department on May 6, 2024. The patient could not verbally report symptoms, and all medical decisions were signed by his family surrogates based on the patient’s best interests.

He had multiple chronic comorbidities across cardiovascular, respiratory, urinary, and neurological systems: long-standing hypertension and coronary artery disease managed with oral amlodipine besylate 2.5 mg daily and atorvastatin calcium 20 mg nightly; chronic bronchiectasis with emphysematous bullae and chronic Pseudomonas aeruginosa airway colonization; bladder diverticulum with calculi; and mild cerebral atrophy. He had no history of diabetes, cerebrovascular accidents, renal disease, major trauma, infectious diseases such as hepatitis or tuberculosis, or any known drug or food allergies. His surgical, transfusion, and vaccination histories were unremarkable.

Baseline CGA confirmed high frailty risk: ECOG performance status 2, intact basic activities of daily living (ADL) but severely impaired instrumental ADL, NRS2002 score 2 with mild nutritional risk, and an age-adjusted Charlson Comorbidity Index (aCCI) score indicating high chemotherapy toxicity risk. The CARG (Cancer and Aging Resilience Group) toxicity prediction model estimated an over 85% risk of grade 3 or higher severe adverse events under platinum-doublet chemotherapy. Given this objective frailty stratification, the family declined platinum-doublet intensive chemotherapy, and a low-toxicity sequential regimen with continuous ADT was adopted.

### 2.2 Clinical findings

#### 2.2.1 Physical examination and laboratory tests

The patient presented with poor general status and bilateral pulmonary crackles on auscultation; digital rectal examination detected a nodular enlarged prostate with suprapubic tenderness suggestive of bladder calculi-related urinary irritation. Baseline laboratory tests revealed elevated total prostate-specific antigen (TPSA) at 24.22 ug/L (reference <4.0 ug/L) and pro-gastrin-releasing peptide (ProGRP) at 89.1 pg/mL (reference <69.2 pg/mL) on May 7, 2024. Serial changes in ProGRP, SCCA, and TPSA during the treatment course are shown in **Figure 1**. Cytokeratin 19 fragment (CYFRA21-1) was within normal limits. Mild inflammatory elevation and borderline nutritional indexes were noted, with normal hepatic and renal function. Initial sputum culture was positive for Pseudomonas aeruginosa.

**Figure 1:**
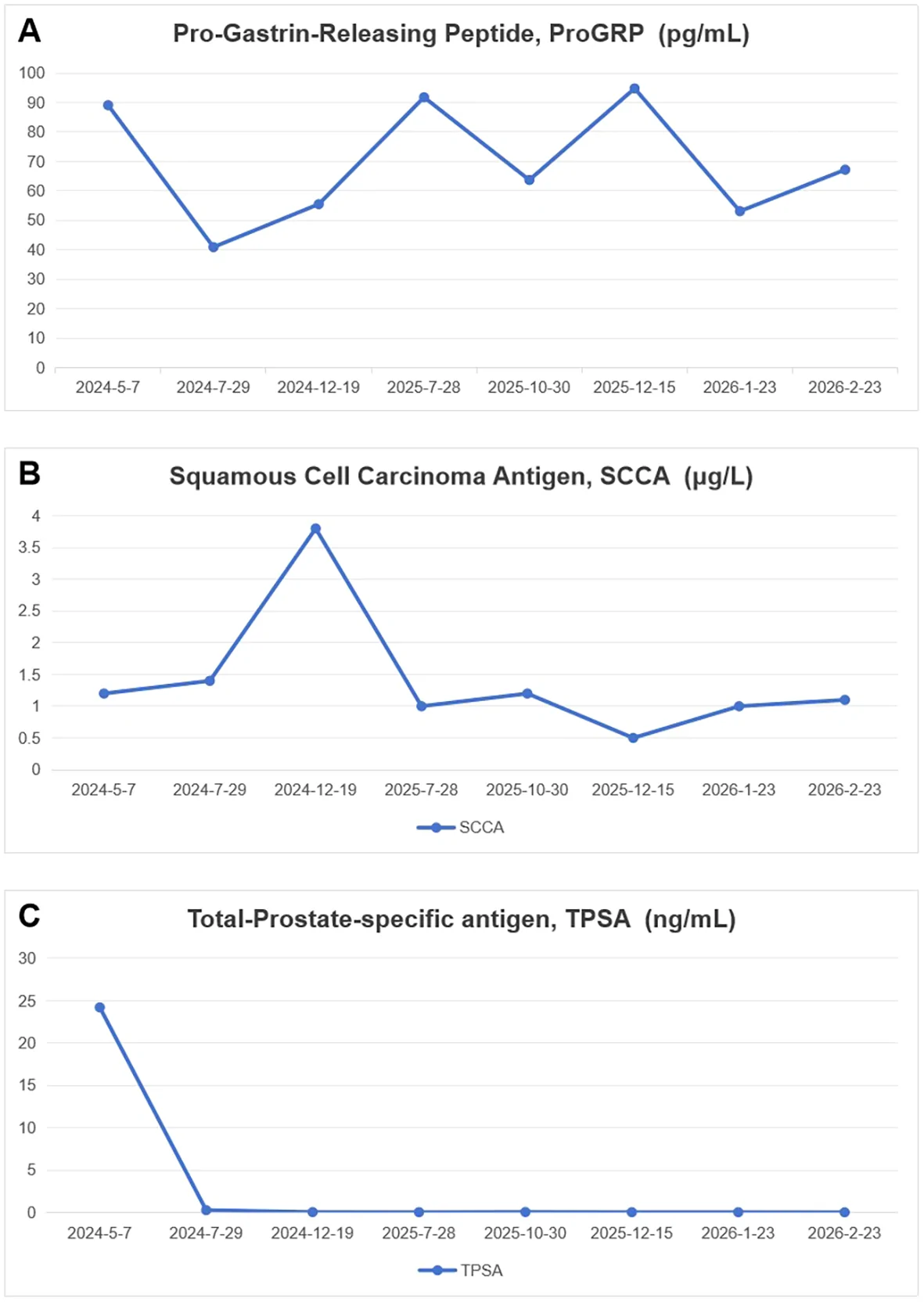
Curves of changes in the patient’s serological indicators. **(A)** Curve of changes in Pro-gastrin-releasing peptide (ProGRP). **(B)** Curve of changes in Squamous cell carcinoma antigen (SCCA). **(C)** Curve of changes in Total prostate-specific antigen (TPSA).

#### 2.2.2 Imaging

A chest contrast-enhanced CT scan on May 3, 2024, identified a solid nodule in the anterior segment of the right upper lobe measuring 14 mm x 13 mm, which had increased in size compared to a prior scan from October 17, 2023. The lesion exhibited lobulation and spiculated margins with post-contrast enhancement, raising high suspicion for malignancy. The scan also revealed findings consistent with chronic pulmonary inflammation, bronchiectasis with possible infection in the right lower lobe, mucus plugging, pulmonary emphysema with multiple bullae, atherosclerotic changes, and right pleural thickening. A subsequent whole-abdomen contrast-enhanced CT on May 9, 2024, noted an enlarged prostate with multiple nodules, prompting further evaluation. Abdominal CT also showed bladder diverticula with calculi, increasing the risk of subsequent urinary retention.

#### 2.2.3 Histopathology

A definitive diagnosis was established via histopathological examination of biopsies obtained on May 11, 2024. A CT-guided lung biopsy of the pulmonary lesion confirmed poorly differentiated squamous cell carcinoma based on immunohistochemistry (IHC): CK(+), P40(+), TTF-1(-), with a Ki-67 index of approximately 20%. The available pathology report documented PD-1 staining with a mouse monoclonal antibody (clone MX033; Fuzhou Maixin Biotechnology Co., Ltd.) and PD-1 positivity in no more than 1% of infiltrating lymphocytes; HER2 was 0. This is distinct from tumor-cell PD-L1 testing. No tumor-cell PD-L1 assay or TPS result was available because the original 2024 slides had been archived. Concurrently, a transrectal ultrasound-guided prostate biopsy (9 cores) revealed multifocal prostatic adenocarcinoma. The Gleason score was 3 + 4 = 7 (Grade Group 2) in the left apex (60% involvement) and 3 + 3 = 6 (Grade Group 1) in several other cores. The two lesions had distinct histologic features and IHC profiles, verifying synchronous independent primary cancers and ruling out cross-organ metastasis.

### 2.3 Diagnostic assessment

Based on the integration of clinical presentation, imaging findings, serum tumor markers, and definitive histopathology, the patient was diagnosed with synchronous double primary malignancies. Combined with the patient’s clinical manifestations, imaging characteristics, and biological behavior of the lesion, the pulmonary tumor was clinically staged as Stage I (T1N0M0); the lesion size (14 mm) conformed to the definition of T1 stage (tumor maximum diameter <=3 cm). The prostate adenocarcinoma was staged as Stage II (T2N0M0). Additional diagnoses included hypertension, coronary atherosclerotic heart disease, pulmonary emphysema with bullae, bronchiectasis, bladder diverticulum with calculus, and mild cerebral atrophy.

Differential diagnoses for the pulmonary space-occupying lesion included pulmonary tuberculosis, benign pulmonary tumor, and metastatic tumor. Combined with elevated tumor markers, typical malignant imaging manifestations, and pathological biopsy results, primary lung squamous cell carcinoma was confirmed. For prostate lesions, elevated PSA and multiple prostatic nodules highly suggested prostate cancer, which was finally verified by puncture pathology. Considering the two tumors occurred simultaneously with independent pathological origins, synchronous double primary cancers were diagnosed. The patient had multiple comorbidities and poor physical tolerance, with a relatively poor long-term prognosis.

### 2.4 Therapeutic intervention

Given the patient’s frailty and the family’s decision to decline platinum-doublet intensive chemotherapy, a three-line sequential low-toxicity regimen was designed with continuous ADT for prostate cancer throughout all treatment phases. Before systemic treatment, surgery and radiotherapy/stereotactic body radiotherapy (SBRT) were considered. Surgery was not pursued because of the patient’s frailty, significant cardiopulmonary comorbidities, and chronic structural lung disease. Radiotherapy/SBRT required reproducible immobilization and repeated treatment cooperation; this was not feasible for this congenitally deaf-mute patient with severe communication limitations despite surrogate communication and graphic explanation. After discussion, the family also declined local treatment.

#### 2.4.1 First-line therapy (May-Aug 2024: ADT + afatinib)

Combined androgen blockade with leuprorelin microspheres 3.75 mg subcutaneously every 4 weeks, abiraterone acetate 1 g orally daily, and prednisone 5 mg orally twice daily was administered for prostate cancer. For LUSC, first-line targeted therapy with oral afatinib was initiated. Oral targeted therapy was prioritized for convenient home care for this deaf-mute patient dependent on family caregivers. The patient’s hypertension and dyslipidemia continued to be managed with his existing medications.

#### 2.4.2 Second-line therapy (Aug-Dec 2024: single-agent oral vinorelbine)

In August 2024, afatinib was withdrawn due to suspected bladder involvement and drug-induced urinary retention. Consistent with baseline records and abdominal CT findings, the patient had pre-existing mechanical obstruction from prostatic nodules and bladder diverticular calculi. Afatinib’s mucosal irritating side effects aggravated lower urinary tract congestion and triggered urinary outlet obstruction. Vinorelbine was then administered orally at 20 mg twice weekly. This non-standard schedule was selected after discussion with the family because of the patient’s frailty, outpatient-care needs, and concern about treatment tolerability; ADT was maintained. However, by December 2024, follow-up imaging confirmed disease progression of the lung cancer to Stage IV (T2N2M1), with an increase in the primary lesion size and new pleural, nodal, and bony metastases. The CT images documenting the initial and progression stages are shown in **Figure 2**.

**Figure 2:**
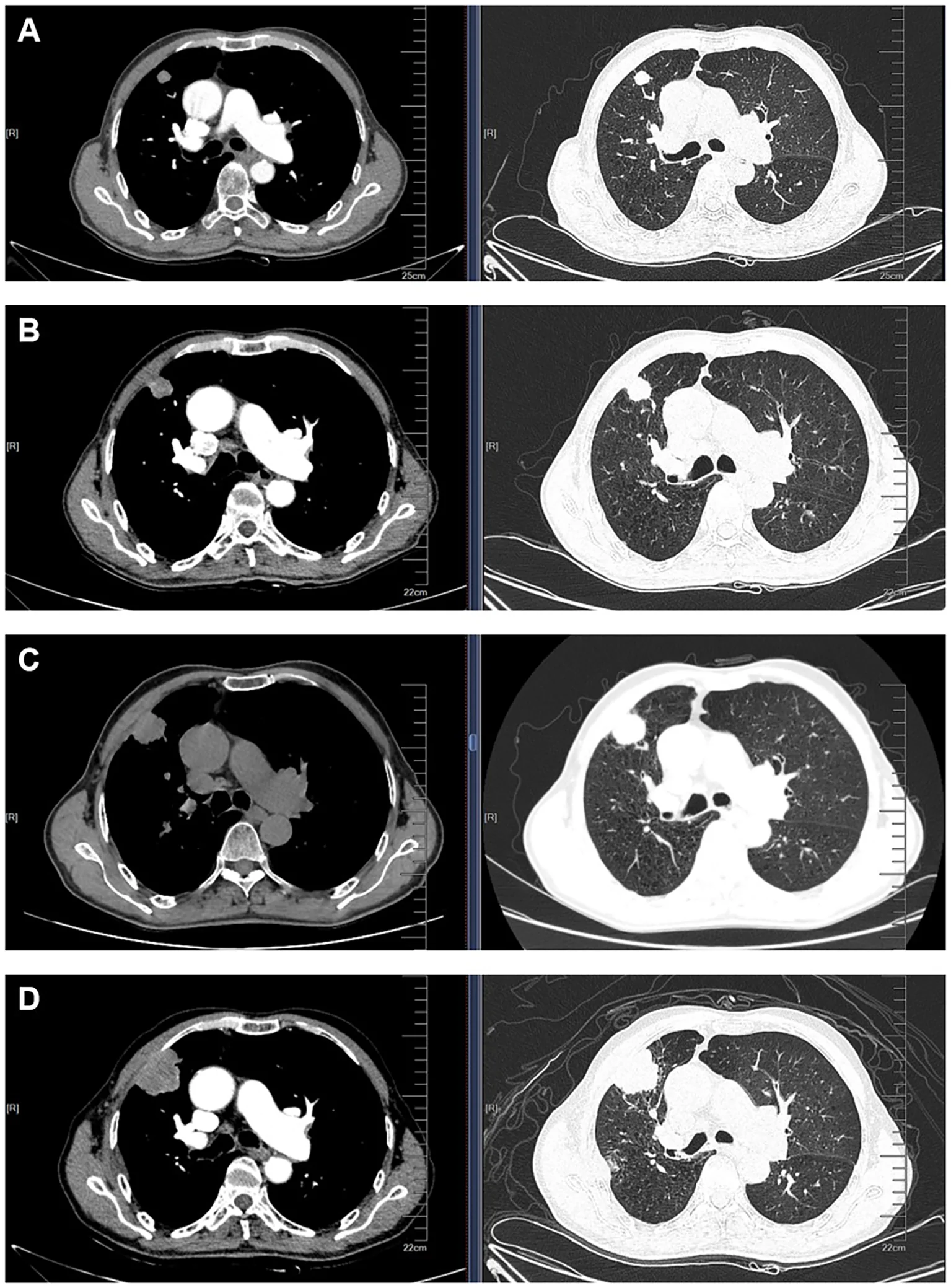
CT images of the patient at different stages (Phase 1). **(A)** May 3, 2024. **(B)** August 1, 2024. **(C)**. October 7, 2024. **(D)** December 20, 2024.

#### 2.4.3 Third-line therapy (Dec 2024-Jun 2026: standard-dose sintilimab + ADT + bone protection)

After detailed communication regarding immunotherapy efficacy and immune-related adverse event risks with the patient’s family surrogates, sintilimab 200 mg intravenously was initiated as third-line monotherapy for advanced LUSC on December 24, 2024. Although the intended schedule was every 21 days, treatment was not uninterrupted. A total of 12 administrations were recorded on December 24, 2024; January 13, February 10, March 10, April 14, July 27, September 5, October 5, and December 21, 2025; and February 3, February 28, and June 9, 2026. All doses were 200 mg, without dose reduction. The deviations from the planned q21-day schedule, including a longest interval of more than 3 months between April 14 and July 27, 2025, were due to recurrent pulmonary infections. Androgen deprivation therapy for prostate cancer was maintained (later switched to goserelin administered every 3 months to reduce outpatient frequency), and bone-targeted therapy with incadronate disodium was added monthly to manage bone metastases. Standardized sputum monitoring plus reserved anti-Pseudomonas antibiotics were arranged to support infection management. The observed dual-tumor control in this single case cannot establish that ADT enhanced PD-1 inhibitor efficacy.

In accordance with the CARE guidelines, a timeline table of the whole treatment process was supplemented to clearly display all treatment nodes, medication schemes, and follow-up time points (**Table 1**).

**Table 1:** Timeline of the patient’s whole treatment and follow-up process.

Time	Tumor stage	Treatment	Lesion size	Infection event	TPSA/ProGRP
May 2024	LUSC T1N0M0 (Stage I); Prostate T2N0M0 (Stage II)	ADT (leuprorelin + abiraterone + prednisone) + afatinib for LUSC	14 mm x 13 mm	Baseline Pseudomonas aeruginosa colonization	TPSA 24.22 ug/L; ProGRP 89.1 pg/mL
Aug 2024	LUSC progression (suspected bladder involvement)	Switched afatinib to vinorelbine; ADT maintained	Enlarged	Drug-induced urinary retention	TPSA suppressed; ProGRP elevated
Dec 24, 2024	LUSC Stage IV (T2N2M1) with pleural, nodal, bony metastases	Sintilimab 200 mg IV initiated; intended q21-day schedule; ADT maintained; incadronate added for bone metastases	43 mm x 32 mm	No acute infection	TPSA suppressed; ProGRP elevated
Jan-Apr 2025	LUSC partial response	Sintilimab 200 mg IV on Jan 13, Feb 10, Mar 10, and Apr 14; temporary interruption thereafter for recurrent pulmonary infection	Shrinking	Bronchiectasis exacerbation (P. aeruginosa)	Markers declining
Jul-Sep 2025	LUSC partial response	Sintilimab 200 mg IV on Jul 27 and Sep 5 after infection-related delay; ADT maintained	Further shrinkage	Bronchiectasis exacerbation (P. aeruginosa)	Markers declining
Oct-Dec 2025	LUSC partial response	Sintilimab 200 mg IV on Oct 5 and Dec 21; intermittent infection-related delays; ADT maintained	Further shrinkage	Bronchiectasis exacerbation (P. aeruginosa)	Markers stable
Feb 2026	LUSC durable partial response	Sintilimab 200 mg IV on Feb 3 and Feb 28; ADT maintained	14 mm x 9 mm	Bronchiectasis exacerbation (P. aeruginosa)	Markers stable
Jun 2026	Clinical and tumor status reported stable	Sintilimab 200 mg IV on Jun 9; total 12 administrations from Dec 24, 2024 to Jun 9, 2026; treatment was not uninterrupted	N/A	No acute infection recorded at latest treatment	N/A

### 2.5 Follow-up and outcomes

Follow-up lasted from December 2024 to Jun 2026, with outcomes categorized into anti-tumor efficacy, comorbidity complication control, and immunotherapy safety.

#### 2.5.1 Dual tumor efficacy

Long-term partial response was achieved for lung squamous carcinoma: the primary lesion shrank from 43 mm x 32 mm to 14 mm x 9 mm, with complete regression of pleural, lymphatic, and bone metastases. Prostate adenocarcinoma maintained stable disease under continuous ADT, with sustained TPSA suppression. Tumor marker trends were consistent with imaging improvement. The serial CT images obtained during the response phase are shown in **Figure 3**.

**Figure 3:**
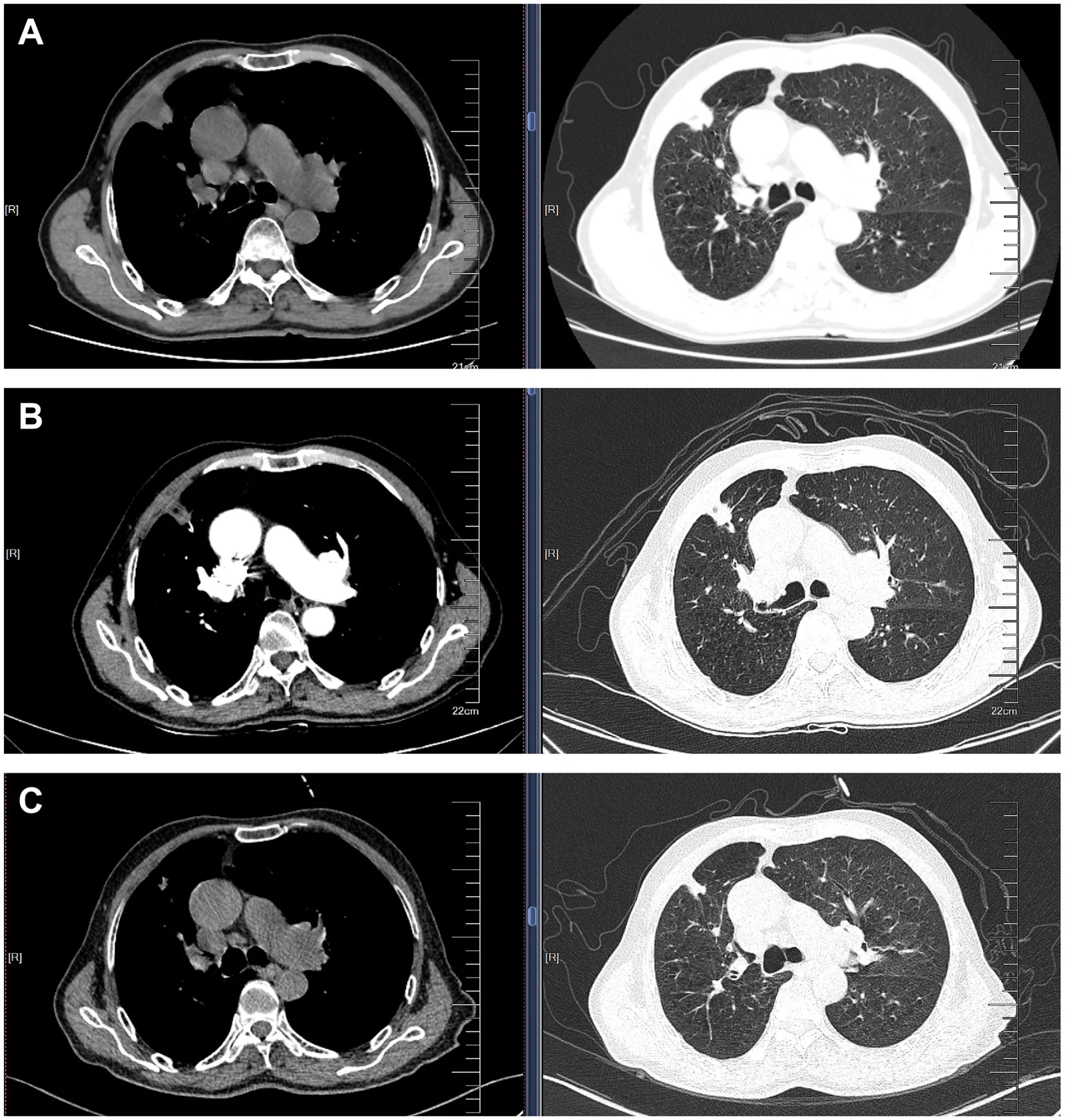
CT images of the patient at different stages (Phase 2). **(A)** April 14, 2025. **(B)** July 29, 2025. **(C)**. February 24, 2026.

#### 2.5.2 Recurrent bronchiectasis infection management

Five clinically documented Pseudomonas aeruginosa infectious exacerbations occurred in April, July, October, and December 2025 and January 2026, against a background of chronic airway colonization. Targeted culture-guided antibiotics (including cefoperazone-sulbactam and piperacillin-tazobactam) were administered when clinically indicated, and sintilimab was temporarily suspended rather than permanently discontinued. To reconcile the actual treatment exposure, 12 standard-dose administrations were delivered from December 24, 2024 to June 9, 2026, not seven uninterrupted cycles over 13.5 months. The longest interval exceeded 3 months, and treatment interruptions were attributed to recurrent pulmonary infection. At the latest recorded follow-up, the patient was clinically stable and the tumor was reported as stable.

#### 2.5.3 Long-term urinary outcomes

After discontinuing afatinib, drug-induced urinary retention did not recur throughout follow-up. Baseline bladder diverticular calculi and prostatic lesions caused no repeated urinary obstruction or secondary infection, confirming the benefit of selecting anti-tumor agents without urological mucosal toxicity.

#### 2.5.4 Safety of full-dose sintilimab

All adverse events were graded by CTCAE v5.0 with systematic organ-by-organ surveillance. To strengthen the safety description of standard-dose sintilimab, irAEs were evaluated after each administered treatment cycle (**Table 2**). No grade 2 or higher immune-related adverse events were documented during the reported follow-up.

**Table 2:** Systematic irAE grading by organ system (CTCAE v5.0) during full-dose sintilimab therapy.

Organ System	irAE Type Monitored	Highest Grade Observed	Management
Endocrine	Thyroid dysfunction (hyper/hypothyroidism), adrenal insufficiency	Grade 0	No intervention required; thyroid function monitored each cycle
Pulmonary	Immune-mediated pneumonitis	Grade 0	No pneumonitis; recurrent infectious exacerbations distinguished from irAE and treated with culture-directed antibiotics
Skin	Rash, pruritus, dry skin	Grade 1 (transient dry skin)	No intervention; self-resolved
Gastrointestinal	Colitis, diarrhea	Grade 0	No colitis or diarrhea observed
Hepatic	Immune-mediated hepatitis	Grade 0	Liver function monitored each cycle; no elevation
Renal	Immune-mediated nephritis	Grade 0	Renal function stable throughout
Hematologic	Leukopenia, thrombocytopenia	Grade 1 (mild leukopenia during acute infection only)	Normalized after antibiotics; unrelated to sintilimab
Neurologic	Neuropathy, myasthenia	Grade 0	No neurologic irAE observed
Musculoskeletal	Myalgia, arthralgia	Grade 0	No musculoskeletal irAE observed
Cardiac	Immune-mediated myocarditis	Grade 0	No cardiac irAE; baseline coronary disease stable

During the reported follow-up after initiation of standard-dose sintilimab, systematic irAE monitoring showed no grade 2 or higher immune-related adverse events in this frail multi-comorbidity elderly patient. Mild leukopenia only appeared during acute pulmonary infectious episodes and normalized after antibiotics, unrelated to sintilimab. Only grade 1 transient dry skin was observed without intervention.

#### 2.5.5 Overall summary

The combination of standard-dose sintilimab and continuous androgen deprivation therapy was associated with sustained synchronous disease control for the patient’s two distinct primary malignancies. The lung squamous cell carcinoma maintained durable partial response with marked shrinkage of the primary lesion and complete resolution of distant metastases, while prostate adenocarcinoma remained stable under long-term hormonal suppression. Standardized tiered infection management controlled recurrent bronchiectasis exacerbations via culture-targeted antibiotics and temporary immunotherapy interruption, without permanent treatment discontinuation. The treatment schedule was intermittent rather than continuous: 12 administrations were delivered between December 24, 2024 and June 9, 2026. No grade 2 or higher immune-related adverse events were documented during the reported follow-up.

#### 2.5.6 Patient-reported experience and quality of life

As a congenitally deaf-mute patient unable to verbally describe subjective discomfort, all quality-of-life feedback and symptom perception were collected through family caregivers and standardized visual symptom scales. Before immunotherapy, the patient suffered frequent severe cough, chest and bone pain, poor sleep, and limited daily mobility, which greatly impaired basic self-care ability. After initiating standard-dose sintilimab combined with ADT, tumor-related pain and respiratory distress gradually relieved. The patient recovered stable daily activity, maintained consistent ECOG PS 2, and could independently complete eating, dressing, and short-distance walking. Treatment delivery included multiple infection-related pauses rather than uninterrupted q21-day dosing. Surrogates confirmed the patient’s stable mental state and better tolerance to long-term outpatient management.

## 3 Discussion

### 3.1 Core clinical dilemmas of this complex frail patient cohort

This case presents four distinct, rarely reported clinical characteristics that together highlight an unaddressed clinical need in geriatric oncology: (1) a frail elderly patient with multiple chronic cross-system comorbidities, including bronchiectasis with chronic Pseudomonas aeruginosa colonization and bladder diverticular calculi, where standardized CGA and aCCI scoring objectively confirmed high frailty risk and the family declined platinum-doublet intensive chemotherapy; (2) congenital deaf-mutism resulted in complete loss of verbal communication, requiring full proxy informed consent and non-verbal symptom assessment throughout the entire treatment process; (3) synchronous dual primary malignancies, namely prostate adenocarcinoma and LUSC, with disease progression after first-line afatinib and second-line vinorelbine treatment; and (4) observed long-term tumor control during standard-dose sintilimab with continuous ADT and an infection-management strategy that used temporary rather than permanent immunotherapy discontinuation.

### 3.2 Hypothesis-generating interpretation of sintilimab combined with sustained ADT

Faced with the above multi-dimensional clinical bottlenecks that lack unified treatment guidelines, a sequential low-toxicity regimen with standard-dose sintilimab combined with continuous ADT was adopted. The following discussion is a hypothesis-generating interpretation of this single patient’s clinical course and does not establish causality or a synergistic anti-tumor mechanism.

First, the observed response of the LUSC occurred while the patient received continuous ADT for concurrent prostate adenocarcinoma. Experimental and translational studies suggest that androgen deprivation may alter components of the tumor immune microenvironment, including cytokine signaling and immune-cell infiltration. However, this case did not include serial tumor immune profiling, and a tumor-cell PD-L1 TPS was unavailable. It is therefore not possible to conclude that ADT remodeled the tumor immune microenvironment, overcame PD-L1 negativity, or caused the observed lung-tumor response. Sintilimab and ADT have distinct therapeutic targets and were clinically co-administered in this patient; their apparent compatibility and the observed dual-tumor control should be regarded as a hypothesis-generating observation requiring validation in prospective studies.

Second, this patient received the standard 200 mg dose of sintilimab without dose reduction, but actual dosing was intermittent because of recurrent pulmonary infection. The absence of documented grade 2 or higher immune-related adverse events during the reported follow-up is reassuring for this individual patient; it does not verify safety or efficacy of standard-dose PD-1 immunotherapy for all frail elderly patients with multiple comorbidities. Comprehensive pre-treatment frailty assessment and supportive management may help guide individualized decisions, while the role of dose modification should be determined case by case rather than inferred from a single report.

Third, disease progression after first-line afatinib and second-line oral vinorelbine prompted consideration of sintilimab plus ongoing ADT. Initial oral afatinib was discontinued after urinary retention in the setting of pre-existing bladder diverticular calculi and prostatic mechanical obstruction. Vinorelbine 20 mg orally twice weekly was selected as an individualized, non-standard regimen because of frailty and outpatient-care considerations; subsequent imaging nevertheless showed progression. The family had declined platinum-doublet intensive chemotherapy after review of the high predicted toxicity risk. In this constrained setting, standard-dose sintilimab plus sustained ADT was selected as a feasible salvage option. The observed outcome cannot demonstrate superiority over alternative regimens or establish a unique clinical advantage of immune-endocrine combination therapy.

### 3.3 Standardized management of chronic comorbidity complications

The patient’s baseline bronchiectasis with persistent Pseudomonas aeruginosa colonization and bladder diverticular calculi combined with prostatic lesions constituted two major treatment barriers. For recurrent bronchiectasis exacerbations during sintilimab therapy, sputum surveillance and culture-guided anti-pseudomonal antibiotics were implemented. Sintilimab was temporarily suspended during acute infection control and resumed after clinical improvement; this individualized approach may be considered in similar circumstances but requires clinical judgment. The urinary adverse event after afatinib also suggests that pre-treatment urinary assessment may be useful for elderly men with prostate lesions and bladder calculi. Because this is a single case, the proposed supportive-care approach should not be interpreted as a universal protocol.

### 3.4 Limitations

As a unique feature of this case, congenital deaf-mutism deprived the patient of verbal symptom feedback. Routine symptom inquiry and pain assessment could not be implemented; therefore, picture-based evaluation scales and family surrogates were adopted to monitor cough, dyspnea, urinary discomfort, and immune-related adverse events. All informed consent documents were explained via graphic demonstration and signed by legal family representatives. For patients with communication dysfunction complicated with frail status and multiple malignancies, standardized proxy assessment and consent procedures should be established to avoid missed identification of treatment-related complications. We also acknowledge the inherent limitations of a single-case report, which precludes broad generalization of the findings. The long-term efficacy, optimal timing, and duration of sintilimab in the specific context of dual prostate and lung cancers remain undefined and warrant further investigation.

### 3.5 Clinical implications and future research

This case suggests several considerations for frail elderly patients with synchronous lung and prostate malignancies, bronchiectasis, bladder calculi, and communication disorders. Pre-treatment standardized CGA may help stratify frailty and inform the selection of lower-toxicity sequential regimens (5). For selected patients who decline platinum-doublet intensive chemotherapy after targeted therapy and individualized single-agent chemotherapy, standard-dose sintilimab combined with sustained ADT may be considered as a salvage option; evidence from this single case is insufficient to recommend it routinely. For recurrent bronchiectasis infection during immunotherapy, culture-directed antibiotics plus temporary treatment interruption may support ongoing therapy in selected patients. Visual proxy assessment and standardized proxy informed consent are important for congenitally deaf-mute patients. Prospective studies are needed to evaluate efficacy, safety, optimal timing, and predictive biomarkers.

Clarification of immunohistochemical indicators: The available original pathology report documented PD-1 staining with a mouse monoclonal antibody (clone MX033) in infiltrating lymphocytes, with positivity in no more than 1%. PD-1 is expressed on immune cells and is distinct from PD-L1 expression on tumor cells. The archived 2024 slides were not available for retrospective tumor-cell PD-L1 testing, and no tumor proportion score (TPS) was available. Accordingly, this manuscript does not classify the lung squamous cell carcinoma as PD-L1-negative and does not draw conclusions based on a tumor-cell PD-L1 result. This distinction is now stated in the histopathology section, abstract, discussion, and **Figure 4** legend.

**Figure 4:**
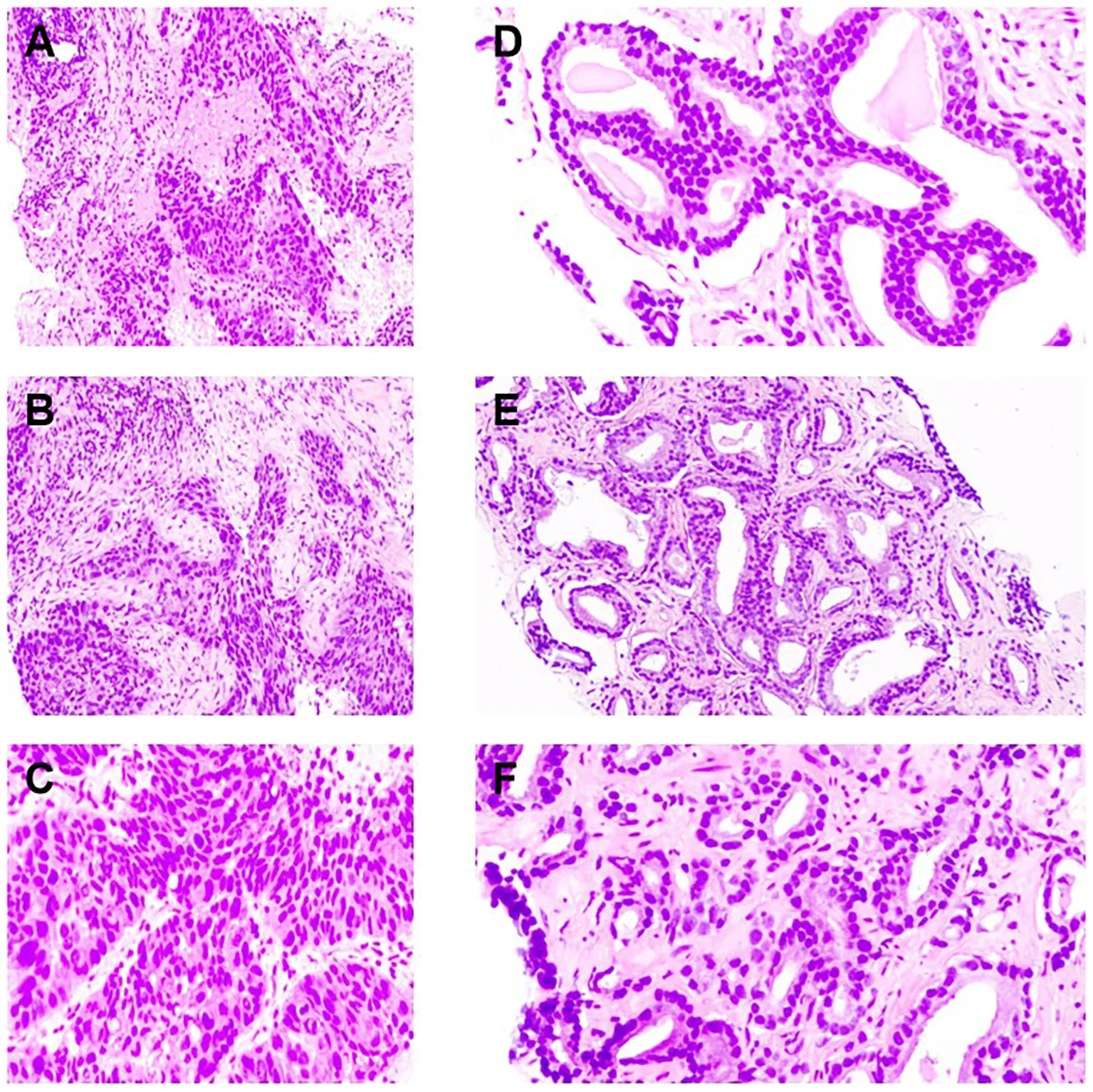
Patient histopathological report findings. **(A-C)** (Lung Tissue) Immunohistochemistry Results: The tumor cells were positive for CK (+), negative for CK7 (-), negative for TTF-1 (-), positive for P40 (+), negative for Syn (-), negative for CgA (-), negative for CD56 (-), and positive for INSM1 (+). The Ki-67 proliferation index was approximately 20% (+). The available pathology report documented PD-1 staining with a mouse monoclonal antibody (clone MX033; Fuzhou Maixin Biotechnology Co., Ltd.) in infiltrating lymphocytes, with positivity in no more than 1%. This is not a tumor-cell PD-L1 assay; no tumor-cell PD-L1 TPS result was available. Human epidermal growth factor receptor 2 (HER2) status was 0. **(D-F)** (Prostate Tissue): 1. Transrectal Prostate (Upper Left): Prostatic adenocarcinoma, Gleason score: 3 + 4 = 7, Grade Group: 2. The carcinoma comprised approximately 60% of the submitted tissue. 2. Transrectal Prostate (Mid Left): Prostatic adenocarcinoma, Gleason score: 3 + 3 = 6, Grade Group: 1. The carcinoma comprised approximately 80% of the submitted tissue. 3. Transrectal Prostate (Lower Left): Prostatic adenocarcinoma, Gleason score: 3 + 3 = 6, Grade Group: 1. The carcinoma comprised approximately 40% of the submitted tissue. 4. Transrectal Prostate (Upper Right, Outer): Prostatic adenocarcinoma, Gleason score: 3 + 3 = 6, Grade Group: 1. The carcinoma comprised approximately 10% of the submitted tissue. 5. Transrectal Prostate (Mid Right, Outer): Prostatic adenocarcinoma, Gleason score: 3 + 3 = 6, Grade Group: 1. The carcinoma comprised approximately 20% of the submitted tissue. 6-9. Transrectal Prostate (remaining cores): Prostatic tissue, no carcinoma identified.

## 4 Conclusion

This case reports a frail congenitally deaf-mute elderly patient with synchronous lung squamous cell carcinoma and prostate adenocarcinoma, bronchiectasis with chronic Pseudomonas aeruginosa colonization, and bladder diverticular calculi. During a clinically complex course in which the family declined platinum-doublet intensive chemotherapy, standard-dose sintilimab combined with continuous ADT was associated with durable synchronous tumor control. Because this is a single case with intermittent, infection-related treatment delays and no tumor-cell PD-L1 TPS, it cannot establish causality, mechanism, or biomarker-specific efficacy. Temporary immunotherapy suspension with culture-directed antibiotics supported continued treatment in this individual patient. This hypothesis-generating clinical observation may provide a practical reference for future research and individualized care.

In conclusion, this case suggests that standard-dose PD-1 inhibitor immunotherapy combined with ongoing androgen deprivation may be a feasible individualized strategy for an elderly patient with synchronous prostate adenocarcinoma and LUSC when platinum-doublet intensive chemotherapy is declined. The observed synchronous control of both cancers, achieved during intermittent rather than uninterrupted treatment delivery, should be interpreted cautiously. The report does not establish a causal immune-endocrine interaction or a PD-L1-related mechanism, particularly because a tumor-cell PD-L1 TPS was unavailable. Meticulous management of pre-existing comorbidities, including surveillance and prompt treatment of infectious complications in structural lung disease, was important for the sustained delivery of therapy in this patient. Prospective studies are required before generalization.

## Data Availability

The original contributions presented in the study are included in the article/**Supplementary Material**. Further inquiries can be directed to the corresponding author.

## Supplementary material

Supplementary Table Timeline of the patient’s whole treatment and follow-up process (added as required by CARE guidelines, see **Table 1**).
