# Supplementary material for: Successful management of synchronous prostate adenocarcinoma and lung squamous cell carcinoma with sintilimab and ADT in a frail elderly patient with bronchiectasis: a case report
Source: Front Immunol. 2026 Oct 1;17:1845049. doi: 10.3389/fimmu.2026.1845049 (PMC13669153; doi:10.3389/fimmu.2026.1845049)
Supplement: Supplementary file 1 [file Table1.docx]

# Supplementary Table 1

Timeline of the patient’s whole treatment and follow-up process.

| **Time** | **Tumor stage** | **Treatment** | **Lesion size** | **Infection event** | **TPSA/ProGRP** |
| --- | --- | --- | --- | --- | --- |
| May 2024 | LUSC T1N0M0; prostate T2N0M0 | ADT (leuprorelin + abiraterone + prednisone) + afatinib | 14 × 13 mm | Baseline Pseudomonas aeruginosa colonization | TPSA 24.22 μg/L; ProGRP 89.1 pg/mL |
| Aug 2024 | LUSC progression; suspected bladder involvement | Switched afatinib to vinorelbine; ADT maintained | Enlarged | Drug-induced urinary retention | TPSA suppressed; ProGRP elevated |
| Dec 24, 2024 | LUSC Stage IV (T2N2M1) with pleural, nodal, bony metastases | Sintilimab 200 mg IV initiated; intended q21-day schedule; ADT maintained; incadronate added | 43 × 32 mm | No acute infection | TPSA suppressed; ProGRP elevated |
| Jan–Apr 2025 | LUSC partial response | Sintilimab on Jan 13, Feb 10, Mar 10, Apr 14; interruption thereafter | Shrinking | Bronchiectasis exacerbation | Markers declining |
| Jul–Sep 2025 | LUSC partial response | Sintilimab on Jul 27 and Sep 5 after infection-related delay; ADT maintained | Further shrinkage | Bronchiectasis exacerbation | Markers declining |
| Oct–Dec 2025 | LUSC partial response | Sintilimab on Oct 5 and Dec 21; intermittent delays; ADT maintained | Further shrinkage | Bronchiectasis exacerbation | Markers stable |
| Feb 2026 | LUSC durable partial response | Sintilimab on Feb 3 and Feb 28; ADT maintained | 14 × 9 mm | Bronchiectasis exacerbation | Markers stable |
| Jun 2026 | Clinical and tumor status stable | Sintilimab on Jun 9; 12 total administrations from Dec 24, 2024 to Jun 9, 2026; not uninterrupted | N/A | No acute infection at latest treatment | N/A |
